# Impacting tumor cell-fate by targeting the inhibitor of apoptosis protein survivin

**DOI:** 10.1186/1476-4598-10-35

**Published:** 2011-04-06

**Authors:** Ronan J Kelly, Ariel Lopez-Chavez, Deborah Citrin, John E Janik, John C Morris

**Affiliations:** 1 Medical Oncology Branch, Center for Cancer Research, National Cancer Institute, Bethesda, MD 20892, USA; 2 Radiation Oncology Branch, Center for Cancer Research, National Cancer Institute, Bethesda, MD 20892, USA; 3 Metabolism Branch, Center for Cancer Research, National Cancer Institute, Bethesda, MD 20892, USA; 4 Division of Hematology-Oncology, Department of Medicine, University of Cincinnati, Cincinnati, OH 45267, USA

## Abstract

Survivin (*BIRC5*), a member of the inhibitor of apoptosis protein (IAP) family that inhibits caspases and blocks cell death is highly expressed in cancer and is associated with a poorer clinical outcome. Functioning simultaneously during cell division and apoptosis inhibition, survivin plays a pivotal role in determining cell survival. Survivin has consistently been identified by molecular profiling analysis to be associated with higher tumor grade, more advanced disease, abbreviated survival, accelerated rates of recurrence, and chemotherapy and radiation resistance. Survivin's differential expression in cancer compared to normal tissue and its role as a nodal protein in a number of cellular pathways make it a highly flexible therapeutic target, suitable for small-molecule inhibitiors, molecular antagonists, and vaccination-based therapies. By targeting survivin it is hoped that multiple tumor signaling circuitries may be simultaneously disabled. This effect may be applicable to many tumor histologies irrespective of specific genetic makeup. To date, survivin inhibitors have shown modest activity as single agents, but it is anticipated that when given in combination with cytotoxic chemotherapy or monoclonal antibodies they may exhibit enhanced efficacy. This review discusses the complex circuitry of survivin in human cancers and highlights clinical trials involving novel agents that target this important protein.

## Introduction

Survivin (*BIRC5*), is a member of the family of inhibitors of apoptosis proteins (IAPs) [1,2] of which eight members are known, including X-linked inhibitor of apoptosis (XIAP), cIAP_1_, cIAP_2_, NAIP (NLR family, apoptosis inhibitory protein), livin, ILP2 (IAP-like protein 2), BRUCE and survivin [3,4]. Survivin, the smallest family member, is a 142-amino acid, 16.5 kDa protein encoded by a single gene located on the human 17q25 chromosome, consisting of three introns, and four exons [2,5,6] and exists physiologically as a functional homodimer [7,8]. Alternative splicing of survivin pre-mRNA produces five different mRNAs with the potential to encode up to five distinct proteins, survivin, survivin 2B, survivin ΔEx3, survivin 3B and survivin 2α[9-11]. Survivin has been implicated in both control of cell survival and regulation of mitosis in cancer [5,12-14]. Survivin is preferentially and highly expressed in cancer cells, with little expression in most normal non-dividing adult tissues (Table 1) [5]. The integral role of survivin in cancer cell division and survival makes it an attractive therapeutic target to inhibit cancer cell growth [1,2]. It was originally suggested that survivin inhibits cell death induced via the extrinsic and intrinsic apoptotic pathways and confers resistance to apoptosis by directly suppressing caspase activity [14]. Although the exact mechanism of action is unknown, current evidence is that most IAPs, including survivin, block apoptosis by mechanisms other than by direct initiator or effector caspase inhibition [15-17]. Survivin is now thought to function upstream of the effector caspases by inhibiting caspase 9[18], by forming a survivin-hepatitis B X-interacting protein (HBXIP) complex bound to pro-caspase-9 thereby preventing the recruitment of apoptotic protease activating factor 1 (Apaf-1) to the apoptosome [19]. Additionally survivin associates with XIAP enhancing its inhibition of caspase-9 activation [20]. Survivin is inhibited by SMAC/DIABLO (second mitochondria-derived activator of caspases/direct inhibitor of apoptosis binding protein with low pI) which results in the displacement of bound IAPs, which, may then bind to and inhibit caspase function [21,22].

**Table 1:** Over-expression of survivin in common human malignancies

Cancer	Expression (%)
Lung cancer	85.5% [76]

Esophageal cancer	80% [128]

Breast cancer	70.7% - 90.2% [33,129]

Pancreatic cancer	76.9% - 88% [130,131]

Ovarian cancer	73.5% [132]

Malignant melanoma	67% [67]

Colorectal cancer	63.5% [40]

Hepatocellular cancer	41%-87% [133,134]

Gastric cancer	34.5% - 68% [37,135]

Bladder cancer	57.8% [136]

Acute myeloid leukemia	54.8% [137]

Acute lymphocytic leukemia	68.8% [137]

Some investigators have suggested that the primary function of survivin is in controlling cell division, rather than apoptosis inhibition [23,24]. Survivin is up-regulated during cell division and is closely associated with centrosomes and mitotic spindle microtubules. It controls chromosome spindle-checkpoint assembly, thereby ensuring normal cell division. Survivin is maximally expressed during the G_2_M phase of the cell cycle and exists predominantly as a multi-protein complex, known as the chromosomal passenger complex (CPC) [25-27]. By functioning in this complex survivin can facilitate accurate sister chromatid segregation and stabilization of the microtubules in late mitosis [23]. In addition to its direct role in carcinogenesis, survivin may also play a key role in tumor angiogenesis as it is strongly expressed in endothelial cells during the proliferative phase of angiogenesis [12,28,29]. Manipulating the survivin pathway may facilitate endothelial cell apoptosis and promote vascular regression during tumor angiogenesis [29]. Increased expression of survivin also appears to be associated with an increased risk of tumor progression and chemoresistance in many tumor types [30-41]. Results of *in vitro *and *in vivo *studies have shown that survivin down-modulation reduces tumor-growth and sensitizes tumor cells to chemotherapeutic agents such as taxanes, platinum agents, etoposide, gamma-irradiation, and immunotherapy [42]. As an example, resistance to docetaxel is associated with increased levels of survivin [43], and response is often associated with the degree of expression of the various survivin splice variants [44].

### Mechanism of Action of Survivin

Cellular apoptosis is controlled by two pathways. The extrinsic pathway is critical for immune selection and inflammation. It is initiated by the activation of cell death receptors, such as tumor necrosis factor alpha (TNF-α) receptor, located at the cell membrane. The intrinsic pathway is initiated by toxic insult such as radiation or chemotherapy (DNA damaging and anti-microtubule agents) [45]. The two pathways converge at caspase-3 and follow a common process of activating caspases that cause cell death by cleaving essential substrates for cell survival, such as cytoskeletal proteins, DNA repair proteins, and inhibitory subunits of endonucleases [46]. Upon activation of the intrinsic pathway, mitochondrial permeability is increased resulting in the release of both cytochrome C and SMAC/DIABLO. Cytochrome C activates the apoptosome, which in turn activates caspase-mediated proteolysis involved in cell death [47].

SMAC/DIABLO acts as an inhibitor of the IAPs. Upon activation of pro-apoptotic cell signaling, survivin is released from the mitochondria and inhibits caspases-3 and -9. This function requires association with hepatitis B X-interacting protein (HBXIP) and/or with X-linked IAP (XIAP) and is inhibited by SMAC-DIABLO [47]. The regulation of survivin expression and function is complex and occurs at various levels, including transcription, differential splicing, protein degradation, and intracellular sequestration via different ligands [47]. Survivin expression is up-regulated at a transcriptional level by both nuclear factor-κappa β (NF-κB) which in turn, can be activated indirectly by growth factors via the phosphatidylinositol 3-kinase (PI3K)/Akt pathway and by TCF-4/β-catenin pathway [48-50]. The insulin like growth factor-1/mTOR/RAS and Wnt-2 signaling pathways have also been reported to up-regulate survivin via rapid changes in mRNA translation [51-53]. Survivin degradation occurs via the ubiquitin-proteasome pathway in the G_1 _phase of the cell cycle and is stabilized when bound to heat shock protein 90 (Hsp90) [54]. The survivin protein is closely associated with Cdc2/Cdk1 and it is phosphorylated at the threonine-34 (T_34_) residue. This phosphorylation stabilizes the protein and allows it to interact with the mitotic spindle and inhibit caspase-9 [55].

There is accumulating evidence that molecular chaperones play a key role in the regulation of survivin. Binding of survivin to the immunophilin aryl hydrocarbon receptor-interacting protein (AIP) [56] or to heat shock protein-90 (Hsp90) [54] maintains its stability against proteasome dependent destruction. Heat shock protein-60 (Hsp60) has also been identified as a molecular chaperone for survivin [57]. Acute ablation of Hsp60 by small interfering RNA (siRNA) has been shown to destabilize the mitochondrial pool of survivin leading to enhanced mitochondrial dysfunction and caspase-dependent apoptosis. This response involves disruption of the Hsp60-p53 complex, which results in p53 stabilization, increased expression of pro-apoptotic Bax, and subsequent Bax-dependent apoptosis [57].

### Survivin as a Regulator of Cell Division

Survivin plays a central role in cell division, where its expression is coordinated within the cell cycle [2,47]. Survivin levels increase in G_1 _and peak in the G_2_M phase. During mitosis, survivin functions as a regulator of microtubule dynamics and as part of the chromosomal passenger complex (CPC). Survivin functions both at the centrosomes and the microtubules of the metaphase and anaphase spindle providing stabilization and ensuring accurate separation of sister chromatids [2,58,59]. Survivin also localizes to the kinetochores, the mid-region or centromeric portion of the metaphase chromosomes. Here survivin is associated with regulators of cytokinesis, such as Aurora B kinase, the inner centromere protein antigens (INCENP), and Borealin/Dasra [60,61]. This supports the hypothesis that survivin acts as a subunit of the CPC which is required for proper chromosome segregation and cytokinesis [62]. It follows that if survivin is removed from the system, the kinetochore-microtubule system is not properly formed, cell division is either halted or improperly completed, and ultimately cell death occurs. Survivin is also bound to microtubules located in the mitotic apparatus during cell division. Through its association with cyclin-dependent kinase 1 (CDK1), microtubule-bound survivin becomes phosphorylated on Thr34 [1]. This leads to stabilization of the protein and efficient counter-activation of apoptosis in dividing cells. Elimination of survivin leads to apoptosis of dividing cells.

### Survivin Expression in Cancer Cells

Survivin is undetectable in most non-proliferating adult tissues. Exceptions are CD34+ hematopoietic stem cells, placenta, basal cells of the colonic epithelium, and thymus [5]. On the other hand, survivin is over-expressed in a wide variety of cancers (see Table 1) [5]. Overexpression is correlated with advanced disease, accelerated time to recurrence, reduced survival, and resistance to therapy [38]. Survivin has been identified as one of 16 genes within an mRNA expression signature that, in patients with breast cancer, correlates with a poor response to tamoxifen, but a good response to chemotherapy [63]. Furthermore, inhibition of *in vitro *or *in vivo *survivin expression by antisense oligonucleotides, or inhibition of survivin function by *in vitro *inhibition of CDK1-mediated phosphorylation leads to cell death [58]. This is particularly apparent when CDK1 inhibition is combined with taxanes [58]. Recently the mTOR pathway, which can act as a sensor network in stressed conditions, has been implicated in elevating survivin levels [51]. In prostate cancer cells, insulin-like growth factor-1 (IGF-1)-mediated mTOR pathway activation positively modulated survivin levels by increasing the translation of a survivin mRNA pool [51].

#### The role of survivin in malignant melanoma

Genes that are highly expressed in aggressive melanomas includes many with roles in cell cycle regulation/proliferation, DNA replication/repair, and apoptosis pathway-related genes. Survivin is variably expressed in the cytoplasm across the spectrum of melanocytic lesions, with nuclear expression detectable in a subset of malignant melanomas, but not in benign or dysplastic nevi [64]. Nuclear expression has been reported to be an independent predictor for disease recurrence and decreased overall survival in patients with early stage cutaneous melanoma [65]. Patients with nuclear immunoreactivity for survivin showed an increased risk of melanoma recurrence during the first three post-operative years and an increased risk of death. Cytoplasmic survivin staining has not demonstrated a correlation with patient survival [65]. This suggests that the nuclear localization of survivin may play a role in the transformation process. Chen and co-workers demonstrated the up-regulation of survivin in primary melanomas as compared to benign nevi [66]. Similarly, Nasr et al found immune-histochemistry (IHC) nuclear staining for survivin was present in 12 of 18 cases (67%) of malignant melanoma with an average index of 7% (range 0%-15%), and no nuclear staining was present in any benign lesions examined [67]. Expression of survivin, bcl-2, bax, and bcl-X in sentinel lymph node (SLN) biopsies was assessed in 36 patients with stage I and II melanoma using RT-PCR and Southern blotting and correlated to overall survival. Survivin expression correlated with the outcome of patients in a statistically significant way (P < 0.005), whereas the expression of bcl-2, bax, and bcl-X, did not seem to correlate to progression of disease [68]. In this study, 61.5% of patients expressing survivin in the SLN progressed or died of melanoma. In patients negative for survivin expression, 100% were disease-free at a median follow-up time of 52.9 months suggesting that survivin gene expression in SLNs may be a useful prognostic indicator. There was no correlation between *bcl-2*, *bax*, and *bcl-X *gene expression and outcome [68]. Survivin expression has been studied in a limited number of non-cutaneous melanomas. In uveal melanomas, expression of survivin was reported to be low and did not correlate with outcome, resistance of the tumor to brachytherapy, or the presence of liver metastases [69]. Survivin expression has also been reported in a case of esophageal melanoma [70], but its impact is unclear. A phase II study investigated YM155 (section 5.1), a small molecule survivin suppressor as a single agent first line treatment in 34 patients with metastatic melanoma. One patient had a complete response, one had a partial response, and 11 had stable disease^97^.

#### The role of survivin in solid tumors

Survivin expression has been detected in a wide variety of benign and pre-neoplastic lesions including polyps of the colon, breast adenomas, Bowen's disease and hypertrophic actinic keratosis [71]. Survivin has been shown to play an important role in colorectal tumorigenesis by stimulating the transition of adenomas with mild dysplasia into highly dysplastic lesions [72]. This transition was associated with a significant decrease in the apoptotic index (AI) and significant increases in the Ki-67 labeling index (LI) and microvessel density (MVD) (P < 0.001). The expression of survivin inversely correlated with AI and was positively correlated with Ki-67 LI and MVD (P < 0.001). In esophageal cancer, survivin expression has been correlated with a poor prognosis whereby the median survival for patients with high levels of survivin expression was reduced compared to patients with low expression levels [73]. In this study the median survival for patients with advanced esophageal cancer and with high levels of survivin expression was 9.0 months compared to 30.0 months for those patients with low survivin expression (p = 0.0023) suggesting that survivin expression may provide prognostic information. Similarly patients with advanced gastric cancer with survivin-positive tumors have a significantly lower 5-year survival rate compared to patients with survivin-negative tumors (P < 0.05) [74]. In non-small cell lung cancer (NSCLC), survivin is over-expressed in approximately 80% of tumors and its presence is associated with a reduced survival in patients with resected neoplasms [75,76].

Expression of survivin also correlates with resistance to therapy. In prostate cancer cells treated with docetaxel, apoptosis is induced by the binding of survivin to SMAC/DIABLO and to the mitotic spindle [77]. Nakahara et al have reported that YM155 (Section 5.1) induced regression of established hormone refractory prostate cancer (HRPC) xenografts [78]. *In vitro*, docetaxel resistance was reversed in gastric cancer cells when messenger ribonucleic acid (mRNA) expression of survivin was down-regulated by gambogic acid, a survivin inhibitor [43].

Preliminary results are available for a phase II trial investigating 168-hour continuous iv infusion of YM155 (Section 5.1) in patients with metastatic HRPC who have received prior taxane therapy. The primary endpoint of this trial is PSA response rate. Data on 32 patients has been reported and 2 PSA responders were seen indicating YM155 may have some activity in HRPC [79]. An ongoing study is investigating the combination of YM155 and docetaxel in patients with HRPC and preliminary data appear promising. Survivin over-expression has been associated with platinum and taxane-based chemotherapy resistance in a number of solid tumors [80,81]. It is postulated that vascular endothelial growth factor (VEGF) induced angiogenesis may lead to a transient increase in survivin levels [82] suggesting a role for combining survivin inhibitors with anti-VEGF strategies.

#### The role of survivin in hematological malignancies

Survivin expression has been observed in the majority of malignant lymphoproliferative disorders with the exception of chronic lymphocytic leukemia in which it is expressed at levels significantly lower than that found in normal lymphocytes [83]. Different techniques for detecting survivin expression have been used including immunohistochemistry (IHC), immunoblotting, and messenger RNA (mRNA) detection; however, no studies comparing these methods have been performed. Three trials have utilized IHC to determine expression of survivin in diffuse large B cell lymphoma (DLBCL) and one study used mRNA expression. The largest study included 222 patients from four separate randomized trials [31]; however, the interpretation of the effect of survivin expression on outcome is complicated by the fact that different chemotherapy regimens were used in the different trials. Although most regimens contained an anthracycline, usually doxorubicin, or mitoxantrone, some regimens did not. In this report, tumors were characterized as being positive for survivin expression if cytoplasmic staining was demonstrated in 70-90% of cells, and negative if less than 5% of the tumor showed staining. Survivin expression was observed in 60% of the patients' tumors and was found to confer an inferior outcome. Patients whose tumors did not show expression of survivin had a 5-year survival of 54%, whereas only 40% of survivin-positive cases were alive at five years (P = 0.02). The complete remission rate was lower in the survivin-expressing group, but not significantly different from the survivin-negative cases at 61% versus 68%, respectively (P = 0.29). This resulted in a lower event-free survival in the survivin-positive cases, but the difference did not achieve statistical significance (p = 0.09). The survival difference was examined in a multivariate analysis incorporating the International Prognostic Index (IPI) and was found to be independent of the IPI score. In two other reports that included 60 and 39 patients, respectively, the nuclear localization of survivin was associated with an inferior outcome in one, but not the other [84,85]. The relatively small numbers of patients in these studies may have accounted for this disparity. The statistical significance was lost when the patients in the larger study were grouped according to whether they represented a germinal center or non-germinal center DLBCL. There was a trend towards a worse outcome in both subgroups, but the difference did not achieve significance.

Survivin expression has also been evaluated using an RNAse protection assay [86]. Here the authors reported that survivin mRNA expression was increased in 80% of the tumors examined, but it did not impact on overall survival. Survivin protein expression was not simultaneously examined and it is possible that translational regulation could have resulted in a disparity in the mRNA and protein expression levels. A recent report examining the relationship between survivin mRNA and protein levels in patients with HTLV-1-associated adult T cell leukemia/lymphoma (ATLL) found a strong correlation between mRNA levels and protein expression [87].

There has been increasing interest in survivin as a potential therapeutic target in lymphoma. Antisense oligonucleotides (ASO) have been shown to inhibit survivin expression in a number of DLBCL cell lines. These ASO inhibited cell growth *in vitro *and slowed tumor growth in animals [88].

Clinical studies of investigational survivin inhibitors are ongoing in a number of hematological malignancies. There was great enthusiasm for the small molecule imidazolium-based survivin transcriptional inhibitor YM155 (Section 5.1) following the phase I trial as responses were observed in three of five patients with relapsed B cell lymphoma, including two patients with DLBCL [89]. These results were not reproduced in the phase II trial that included 41 patients with relapsed and refractory DLBCL [90]. At the time of the abstract submission, only one of the first 25 patients enrolled and evaluated for tumor response showed an objective response. YM155 was well tolerated with grade 3 or 4 anemia (16%), neutropenia (8%), fatigue (8%), and deep venous thrombosis (8%) as the only events that occurred in more than 4% of the patients. Due to the potential for synergism of YM155 with other agents, additional trials are underway or planned combining YM155 with chemotherapy or monoclonal antibodies including rituximab and alemtuzumab.

Survivin expression may have even greater prognostic significance in patients with T cell lymphoma [50,87,91,92]. High levels of expression have been reported in patients with HTLV-1-associated ATLL. Survivin levels were greater in the aggressive acute subtype of the disease compared with the more indolent chronic form of ATLL. ATLL patients whose tumors expressed high levels of survivin had an inferior outcome, with a median survival time of 6.4 months compared to 18 months in those patients with low survivin levels [93]. These results have been reproduced in a number of other studies and are similar to the results seen in DLBCL. Inhibition of survivin expression in primary ATL cells using shRNA leads to decreases in tumor cell viability.

Anaplastic large cell lymphoma (ALCL), another relatively uncommon T cell neoplasm, is separated into two prognostic groups based on the expression of the anaplastic large cell kinase gene (ALK). The expression of ALK is most frequently the result of a translocation between chromosomes 2 and 5, resulting in a unique fusion of the ALK and nucleophosmin genes, respectively. ALK-positive ALCL has a superior clinical outcome with about 80% of patients cured with current chemotherapy. In contrast, patients with ALK-negative ALCL demonstrate a worse prognosis with a 50% 5-year survival. Survivin expression was examined in ALCL patients by IHC and was found to be frequently positive with primarily cytoplasmic localization [92]. Approximately 63% of ALK-positive ALCL tumors expressed survivin compared with 47% of the ALK-negative ALCL. Absence of survivin expression in both subtypes conferred an improved prognosis with 100% of the ALK-positive, survivin-negative patients alive at 5 years, and 89% of the ALK-negative, survivin-negative patients alive at 5-years. In contrast only 34% of ALK-positive, survivin-positive patients were alive at 5 years. For the ALK-negative group, the 5-year overall survival was 60% for patients with survivin-positive tumors versus 92% for patients with survivin-negative tumors (P = .04). Survivin expression remained an independent adverse prognostic marker in a multivariate analysis that included IPI score.

### Current therapeutic approaches

#### Transcriptional repressors

YM155 (1-(2-Methoxyethyl)-2-methyl-4,9-dioxo-3-(pyrazin-2-ylmethyl)-4,9-ihydro-H-naphtho[2,3-d]imidazolium bromide) (Astellas Pharma Inc) is a small-molecule suppressor of survivin. YM155 interacts specifically with the 269 base pair survivin core promoter region and functions in a cell cycle independent manner as a transcriptional inhibitor. YM155 has demonstrated potent anti-proliferative activity against various cancer cell lines and preferentially induces cell death in tumor cells. Preclinical data has also shown that it has the ability to serve as a radio-sensitizing agent and to potentiate the antitumor activity of various cytotoxic agents including carboplatin and paclitaxel [94,95]. In preclinical experiments, YM155 has shown promising activity in a wide variety of human tumor xenograft models including non-small cell lung cancer (NSCLC) [96].

YM155 has been investigated in six phase I or II clinical trials in solid tumors and non-Hodgkins lymphoma. It has been shown to be well tolerated with the most common toxicities being reported as grades 1-2 in severity and consisting primarily of stomatitis, pyrexia and nausea [97-100]. In a phase I study conducted in the United States, 41 patients received YM155 at doses ranging from 1.8 to 6.0 mg/m^2^/d by a 168-hour (7 days) continuous intravenous infusion every 3 weeks. The maximum tolerated dose (MTD) was determined as 4.8 mg/m^2^. In a second phase I trial conducted in Japan [101], patients with advanced refractory solid tumors were treated with escalating doses of YM155 administered by continuous i.v. infusion for 168-hours in 21-day cycles. Of the 34 patients enrolled, 33 (median age, 59 years) received at least 1 dose of YM155 and the MTD was determined to be 8.0 mg/m^2^/d. The most common adverse reactions judged to be related to YM155 were microalbuminuria, fever, injection-site phlebitis, fatigue, and decreased hemoglobin/anemia, serum albumin, and lymphocyte count.

As monotherapy, YM155 has shown modest antitumor activity in a phase II trial in patients with advanced NSCLC who had failed one or two prior chemotherapies [98,102]. Two partial responses were observed in 37 patients (5.4%) enrolled in this study, where YM155 was administered as a 7 day continuous infusion at 4.8 mg/m^2^/day, repeated every 3 weeks. Fourteen patients (37.8%) achieved stable disease resulting in a disease control rate of 43.2% (95% CI, 27.1% to 60.5%). Median PFS was 1.7 months (95% CI, 1.3 to 2.8 months). Median overall survival was 6.6 months (95% CI, 4 to 12.2 months), with a 1-year survival rate of 35.1%. Treatment was well tolerated with the majority of treatment discontinuations not YM155 related. A phase I/II study evaluating the combination of YM155 in combination with carboplatin and paclitaxel has now been initiated. YM155 is also being investigated in malignant melanoma, HER-2/neu-negative breast cancer, and in combination with docetaxel in hormone refractory prostate cancer (see Table 2).

**Table 2:** Survivin inhibitors currently in phase I/II clinical trials

Mechanism of action	Compound	Clinical Trials	Indication
**Antisense oligonucleotides**	LY2181308	Phase I	AML
		
		Phase II	AML, NSCLC (+docetaxel) and HRPC (+docetaxel)

	Terameprecol (EM-1421)	Phase I	Leukemia
		
		Phase II	AML, NSCLC (+/- docetaxel), HRPC (+docetaxel)
	
**Transcriptional Repressors**		Phase I	Solid tumors
		
	YM155	Phase II	NSCLC(+carboplatin/paclitaxel), HRPC (+docetaxel), HER2 negative breast cancer (+docetaxel) Melanoma

	Vaccine Therapy With Dendritic Cells - Transfected With hTERT-, Survivin- and Tumor Cell Derived mRNA + ex Vivo T Cell Expansion and Reinfusion	Phase I/II	Melanoma
	
	Multipeptide vaccine: PSMA and Survivin	Phase I/II	Prostate cancer
	
	Survivin peptide vaccine	Phase I/II	Solid tumors
	
	Multipeptide Vaccine: telomerase and survivin	Phase I	Breast cancer
	
**Immunotherapy**	Multipeptide vaccine: MART-1, keyhole limpet hemocyanin, recombinant MAGE-3.1, survivin and autologous dendritic cells	Phase I/II	Melanoma
	
	Multipeptide vaccine: MART-1, gp100 and survivin antigens + GM-CSF +/- IL2	Phase I	Melanoma
	
	Stem Cell Transplant, Chemotherapy, and Biological Therapy (CMV N495 peptide, CMV pp65 peptide, hTERT I540/R572Y/D988Y multipeptide vaccine, pneumococcal polyvalent vaccine, survivin Sur1M2 peptide vaccine)	Phase I/II	Multiple Myeloma
	
	Dendritic Cells Transfected With Survivin, hTERT and p53 mRNA	Phase I/II	Breast Cancer or Malignant Melanoma
	
	Survivin and telomerase peptide-pulsed dendritic cells	Phase I/II	Renal Cell Carcinoma

**AML**, Acute myeloid leukemia; **CMV**, cytomegalovirus; **GM-CSF**, granulocyte-macrophage colony stimulating factor; **HRPC**, hormone-refractory prostate cancer; **hTERT**, human telomerase reverse transcriptase; **IL-2**, interleukin-2; **MAGE**, melanoma-associated antigen **NSCLC**, non-small cell lung cancer; **PSMA**, prostate-specific membrane antigen.

Terameprocol (EM-1421, Erimos Pharmaceuticals) is a semi-synthetic small molecule with antitumor activity occurring via selective targeting of Sp1-regulated proteins, including survivin and cyclin dependent kinases (cdc2) that control cell cycle and apoptosis [103,104]. Terameprocol is in clinical development as a site-specific transcription inhibitor in solid refractory tumors and in leukemias.

#### mRNA Inhibitors

LY2181308 (ISIS/Eli Lilly Pharmaceuticals), a novel modified ASO, is a specific inhibitor of survivin. In a phase I trial in which 24 patients were enrolled, LY2181308 showed a safety and pharmacokinetic (PK) profile consistent with previously described ASOs. Side effects were mild to moderate and no grade 3 or 4 toxicities were described at the MTD of 750 mg [105]. Additionally, a pharmacodynamic study has been conducted in 34 patients, including 22 patients with available pre- and post-treatment biopsies. IHC indicated that survivin expression was reduced in the nucleus and cytoplasm in 11 of 17, and 5 of 14 evaluable pairs, respectively. Gene expression analysis indicated a reduction in survivin expression of 20-50% in 11 of 15 evaluable pairs. Analysis of fresh tumor from endobronchial sampling revealed that 2 of 3 NSCLC patients had a near-complete elimination of survivin-positive cells accompanied by an increase in the fraction of cells with a sub-G1 DNA content, consistent with cell death [106]. Furthermore, a human micro-dosing imaging PK study of an ASO with LY2181308 using carbon-11 radiolabeled LY2181308 ([^11^C]LY2181308) was conducted. In this study pharmacokinetic analysis confirmed that biologically active human tumor drug concentrations of [^11^C]LY2181308 can be achieved. LY2181308 therapy saturated normal tissue kinetics and increased tumor uptake of [^11^C]LY2181308 [107]. LY2181308 is currently being evaluated in phase II trials for relapsed and refractory acute myeloid leukemia, prostate cancer and NSCLC (see Table 2). Other approaches for inactivating specific mRNAs that remain predominantly in the preclinical setting involve the use of hammerhead ribozymes [108,109] and small interfering RNAs [110,111].

#### Small molecule survivin inhibitors

Shepherdin is a small molecule inhibitor in early stage clinical development that acts as an antagonist of the survivin-Hsp 90 complex [112]. This molecule is a 5 amino acid peptide that antagonizes the binding between survivin and Hsp-90 as well as acting as a global inhibitor of Hsp90 function via competition with ATP.

### Immunotherapy

Due to its differential expression by tumors, it has been hypothesized that cancer patients may recognize survivin as a "non-self" protein and mount an immune response against it [113]. Phase I trials using survivin-directed autologous cytotoxic T lymphocytes acitvated with survivin-primed dendritic cells or survivin peptides have been performed. Treatment was well tolerated with limited toxicity [114,115]. Wobser et al, described an early example of survivin-based vaccination therapy in a patient with metastatic pancreatic cancer that achieved a complete regression of liver metastasis after receiving a HLA-A2 restricted survivin peptide plus adjuvant [116]. Recently a phase I-II trial of vaccination with HLA-A*0201-restricted peptides from prostate specific membrane antigen (PSMA) and survivin was carried out in 20 prostate cancer patients with biochemical failure after surgery or radiotherapy. The vaccine consisted of two peptides from PSMA (PSMA4-12 and PSMA711-719) and one from survivin (SVV96-104/97M) given by 4 biweekly (priming) and then 4 monthly administrations (boosting). To selectively eliminate regulatory T cells (Tregs) and possibly enhance immunization, the vaccinations were preceded by cyclophosphamide 300 mg/kq, i.v. The vaccine was well tolerated, with 14/20 patients exhibiting a significant although transient PSA decrease. Most patients (19/20) showed a significant increase of SVV96-104/97M-specific T cells, while a response to PSMA was achieved in about half of the patients. Increments of HLA-A*0201/SVV96-104/97M or PSMA711-719 multimer+ CD8+ T cells were induced in 50% and 35% of patients, respectively. However, vaccination-induced Ag-specific T cells displayed limited cross-reactivity with HLA-A*0201+ prostate cancer cells. These results indicate that the vaccine-induced Ag-specific CD8+ T cells had a reduced ability to cross-recognize prostate cancer cells which could explain why PSA control was achieved only transiently [117].

The tolerability, immunogenicity, and clinical efficacy of three survivin peptides restricted to HLA A1, A2 and B35 were addressed in a phase I/II trial which included 79 patients with metastatic melanoma (n = 61), pancreatic (n = 8), cervical (n = 5), colorectal (n = 2), adrenal gland (n = 2), and Merkel cell carcinoma (n = 1) who failed to respond to systemic standard therapy. Peptide vaccination was safe and vaccine-specific immune responses were induced in 50% of the patients. Objective responses and control of disease were essentially restricted to immunological responders. Three complete responses and three partial responses were observed (OR = 7.6%) with the duration of response ranging from 3 to 36+ months [118]. Due to these encouraging results, ongoing Phase II studies are investigating survivin based immunotherapy in patients with advanced pancreatic, colon and cervical carcinomas as well as in melanoma.

### Survivin as a radiosensitizer

Survivin is known to play an important role in the sensitivity of cells to radiation with high survivin levels corresponding to reduced radiation sensitivity [119,120]. In a number of preclinical studies inhibition of survivin expression has been shown to sensitize tumor cells to ionizing radiation [121-124]. Although apoptosis does not play a major role in the response of solid tumors to radiation as a single agent, inhibition of survivin prior to irradiation leads to an increase in apoptosis and reduced tumor cell survival [121]. In addition, survivin inhibition interferes with normal cell cycle progression and DNA repair [125,126]. Consistent with these observations, a number of small molecule inhibitors of survivin have been shown to enhance the cytotoxic effects of radiation [127]. Collectively, these findings provide a strong rationale for the clinical evaluation of survivin inhibitors administered either concurrently or sequentially with therapeutic radiation.

## Conclusion

Over the last decade it has become increasingly clear that inhibitor of apoptosis proteins play an integral role in maintaining cellular homeostasis. In particular, one of these proteins, survivin serves many functions involved in cell survival including complex intracellular signaling, stabilizing mitosis and facilitating cellular adaptation. Much remains to be learned regarding the biology of survivin and other IAPs in terms of how these molecules intersect with other pathways. Survivin is a highly expressed in many different tumors and its expression correlates with advanced disease, poorer survival, and chemotherapy and radiation resistance. Because of the role it plays, survivin is of increasing interest as a potential therapeutic target in cancer. Survivin antagonists may function not as single protein inhibitors, but rather as global pathway inhibitors that may disable multiple signaling circuits in tumors. Clinical trials have highlighted the problems with attempts to correlate survivin expression with clinical outcome. Small sample numbers, non-uniform treatments, the presence of multiple alternatively spliced survivin mRNAs with differing effects on apoptosis and the different methods of detection of survivin, all lead to difficulty in trial interpretation. Further efforts are required to achieve a greater understanding of the biology of survivin and the other IAPs and more effectively exploit strategies that target this protein in cancer.

## Abbreviations

ALCL: anaplastic large cell lymphoma; ALK: anaplastic lymphoma kinase; ATLL: adult T cell leukemia/lymphoma; HTLV-1: human T cell lymphotrophic virus-1; IAP: inhibitor of apoptosis protein; NSCLC: non-small cell lung cancer; VEGF: vascular endothelial growth factor; DLBCL: diffuse large B cell lymphoma; SMAC: second mitochondria-derived activator of caspases; DIABLO: direct inhibitor of apoptosis binding protein with low pI; mRNA: messenger ribonucleic acid; PK: phramacokinetics; TNF-α: tumor necrosis factor alpha; HBXIP: hepatitis B X-interacting protein; XIAP: X-linked IAP; Hsp90: heat shock protein 90; INCENP: inner centromere protein antigens; CDK1: cyclin-dependent kinase 1; CPC: chromosomal passenger complex.

## Conflict of interest

The authors declare that they have no competing interests.

## Authors' contributions

RK: Conception and design, manuscript writing, final approval of manuscript

AL: Manuscript writing, final approval of manuscript

DC: Manuscript writing, final approval of manuscript

JJ: Manuscript writing, final approval of manuscript

JM: Conception and design, manuscript writing, final approval of manuscript
